# Development of a Highly Specific Immunoassay for Residual Venom Detection of the Toxic Jellyfish *Nemopilema nomurai*

**DOI:** 10.3390/toxics13100881

**Published:** 2025-10-16

**Authors:** Yi Wang, Yinuo Liu, Xiaochuan Hou, Ying Ge, Xiao Peng, Fengling Yang, Liang Xiao, Juan Höfer, Fei Wang, Jingbo Chen

**Affiliations:** 1 Faculty of Naval Medicine, Naval Medical University, Shanghai 200433, China; wy0815@smmu.edu.cn (Y.W.); xiaochuanhou@126.com (X.H.); nmuyingge@foxmail.com (Y.G.); xiaopeng57827@163.com (X.P.); yfl1205@smmu.edu.cn (F.Y.); xiaolian@smmu.edu.cn (L.X.); 2 Department of Dermatology Changzheng Hospital, Naval Medical University, Shanghai 200003, China; liuyinuohyd@163.com; 3 Key Laboratory of Biological Defense Ministry of Education, Second Military Medical University, Shanghai 200433, China; 4 Shanghai Key Laboratory of Medical Bioprotection, Second Military Medical University, Shanghai 200433, China; 5 Escuela de Ciencias del Mar, Pontificia Universidad Católica de Valparaíso, Valparaíso 2340000, Chile; juanhofer@gmail.com

**Keywords:** jellyfish, venom, antibody, ELISA, detection

## Abstract

Accurate detection of residual jellyfish venom is crucial for species identification and clinical management post-envenomation. We developed a highly specific immunoassay for *Nemopilema nomurai* venom using polyclonal antibodies (titer: 1:256,000). The established i-ELISA exhibited linear detection (0–20 ng/mL) with low variability (intra-plate CV: 0.77–2.78%; inter-plate CV: 2.25–5.17%). The kit demonstrated remarkable thermal stability (<15% signal decay after 6 days at 37 °C; detectable positivity through Day 9), suggesting >1-year shelf life at 4 °C. It showed significantly higher sensitivity for *N. nomurai* venom than venoms from *Rhopilema esculentum*, *Chrysaora quinquecirrha*, *Cyanea melanaster*, scorpions, or bees (*p* < 0.01). Validation in murine/human skin envenomation models and serum from systemically intoxicated mice confirmed the reproducibility and stability of residual toxins. This study developed a highly sensitive, specific, reproducible, and stable i-ELISA for *Nemopilema nomurai* venom, providing a methodological basis for creating diagnostic kits for marine envenomation.

## 1. Introduction

‌Envenomation by venomous animals is a global public health issue [1,2]. Beyond the acute, lethal toxicity [3] resulting from the direct action of venom, the majority of severely envenomated patients experience secondary complications, such as cytokine storms [4,5] and multi-organ dysfunction [6,7], which can culminate in death. The time from envenomation to death, often spanning from tens of minutes to several hours, defines a critical therapeutic window [8]. Within this period, accurate detection of residual jellyfish venom (venom proteins, peptides or other antigenic components) is crucial for species identification, venom typing, injury assessment, and prognostic [9,10]. This provides a critical foundation for selecting the optimal antivenom or targeted therapies and for implementing precision treatment. Nevertheless, there is currently a widespread lack of clinical recognition of this therapeutic window, coupled with a scarcity of effective diagnostic tools, which severely impedes improvements in treatment outcomes for envenomation [11].

Our understanding of marine envenomation significantly lags behind that of terrestrial envenomation, a knowledge gap compounded by a scarcity of effective diagnostic tools. For instance, jellyfish, which represent one of the greatest marine threats to humans, typically appear in seasonal blooms (e.g., summer) [1,12], presenting a common, widespread, and substantial threat. They are a paradigmatic example of marine envenomation. Jellyfish envenomation can occur without direct contact with the animal, which complicates the timely and accurate identification of the causative species. Furthermore, jellyfish species exhibit marked differences in venom toxicity; stings from most do not cause severe complications and are managed with established treatment protocols [13]. For lethal species, however, misidentification can lead to missing the critical 2- to 48-h therapeutic window, allowing the condition to progress to a severe systemic inflammatory response, multi-organ dysfunction, and potentially death [4,14]. Therefore, the development of diagnostic assays for the venom of medically significant jellyfish, such as *Nemopilema nomurai* [15,16], the primary species responsible for envenomation in Chinese coastal waters, is of critical importance for the diagnosis and clinical management of marine envenomation.

Compared with conventional laboratory detection methods such as high-performance liquid chromatography and mass spectrometry, immunodiagnostic techniques [17,18] based on antigen–antibody interactions offer practical advantages for clinical and field settings, including high sensitivity, specificity, and throughput, and can be readily adapted for rapid on-site detection platforms such as colloidal gold test strips [19]. In this study, using *N. nomurai* as the target species, we first generated polyclonal antibodies against *N. nomurai* venom and subsequently established an indirect enzyme-linked immunosorbent assay (i-ELISA) for the detection of its venom. This assay exhibited strong specificity, high sensitivity, and good stability, which were validated in skin and serum residues after venom exposure. This work provides a methodological foundation for the development of detection kits for jellyfish and other venomous marine organisms.

## 2. Materials and Methods

### 2.1. Jellyfish Collection and Venom Preparation

*N. nomurai* specimens were collected from the Longwangtang area of the Bohai Sea, Dalian, China, collected during summer months (July–August) of 2023, and the tentacles were immediately frozen at –80 °C and transported to the laboratory. According to the previously reported method [20], the tentacles were stirred at 4 °C for 72 h, followed by filtration through a 200-mesh sieve. The filtrate was centrifuged at 4 °C, 1000× *g* for 10 min, and the supernatant was transferred to activated dialysis bags (MD34 MW, 6000–12,000 Da). Dialysis was performed using 1× PBS at 4 °C for 8 h to obtain the *N. nomurai* tentacle extract (TE). The TE concentration was measured as 5 mg/mL by BCA protein concentration assay kit.

### 2.2. Animal Husbandry

Male Institute of Cancer Rsearch (ICR) mice with an average weight of 25 ± 2 g were obtained from Shanghai Jiesijie Experimental Animal Co., Ltd, Shanghai, China. Mice were housed in ventilated cages at 22–23 °C with 55–60% relative humidity and a 12-h light/dark cycle. Water and standard chow pellets were available ad libitum. All animal care and experimental procedures strictly followed the guidelines of the Institutional Animal Care and Use Committee of the Institute of Nutrition and Food Safety, Chinese Academy of Sciences, and were approved by the Institutional Animal Ethics Committee of the Naval Medical University.

### 2.3. Hemolytic Activity Assay

After anesthesia, blood was collected from the orbital sinus of mice using heparinized glass capillary tubes into 1.5 mL heparin-treated centrifuge tubes. The blood was diluted with an equal volume of PBS, centrifuged at 3000× *g* for 5 min at room temperature, and washed repeatedly until the supernatant was clear. One hundred microliters of pelleted erythrocytes were added to 22.2 mL of PBS to prepare a red blood cell suspension. Subsequently, 100 μL of graded concentrations of TE (final concentrations: 2.5, 1.5, 0.75, 0.25, 0.15, or 0.075 mg/mL) were mixed with 100 μL of red blood cell suspension and equal volumes of PBS and saponin (25 g/mL) served as the negative and positive controls, respectively. Following incubation at 37 °C for 30 min, the mixtures were centrifuged at 2000× *g* for 5 min at 4 °C. A 100 μL aliquot of the supernatant was collected, and its absorbance at 415 nm (OD_415_) was measured using a microplate reader. The percentage of hemolytic activity was calculated using the following formula: Hemolytic activity (%) = [(OD_415__experimental group − OD_415__blank group)/(OD_415__control group − OD_415__blank group)] × 100%.

### 2.4. Polyclonal Antibody Production

The prepared TE was diluted to 1 mg/mL and emulsified with an equal volume of Complete Freund’s Adjuvant (CFA; Sigma, St. Louis, MO, USA). Male New Zealand White (NZW) rabbits (2.0 ± 0.5 kg) were immunized via subcutaneous injection at multiple sites (0.2 mL per site). A second immunization was administered two weeks later, followed by booster immunizations every 7 days. For these boosters, TE was emulsified with Incomplete Freund’s Adjuvant (IFA; Sigma). Blood was collected from the central ear artery to monitor the antiserum titer using an indirect ELISA. Once the titer exceeded 1:50,000, a final booster was given. Seven days post-boost, blood was collected for antiserum preparation. For purification, 10 mL of the serum sample was filtered through a 0.45 μm membrane and loaded onto an affinity chromatography column at a flow rate of 40 mL/h; this process was repeated once. The column was then washed with 20 mL of 1× PBS (pH 7.4) at 70 mL/h. After 10 min, a protein detector (HD-4, Shanghai Qingpu Huxi, Shanghai, China) was connected, and the transmittance (T%) was adjusted to 100% during the wash. Finally, the antibody was eluted with 0.2 M glycine solution (pH 2.7) at 40 mL/h and collected. The final concentration of the purified TE polyclonal antibody was determined to be 16.8 mg/mL.

### 2.5. SDS-PAGE Protein Electrophoresis Assay

SDS-PAGE was performed using a 10% resolving gel prepared with the PAGE Gel Fast Preparation Kit (Yamei, Shanghai, China) in accordance with the manufacturer’s instructions. An aliquot of TE (15 μg) was loaded into each well. Protein maker (Beyotime, Shanghai, China) add 10 uL. Electrophoresis was carried out at a constant voltage of 80 V for 30 min, and subsequently at 120 V until the bromophenol blue dye front migrated to approximately 1 cm from the bottom of the gel. Following the run, the gel was carefully removed from the glass plates and stained with Coomassie Brilliant Blue staining solution (Yamei) for 60 min on a horizontal shaker. The gel was then destained with deionized water until a clear background was achieved. Finally, the gel was scanned using a G:BOX Chemi XX6 High-Performance Fluorescence imaging system (Gene Company Limited, Hong Kong, China).

### 2.6. Immunohistochemistry

The dorsal skin of male ICR mice was depilated. Mice in the *N. nomurai* group (*n* = 4) were placed in disposable containers filled with *N. nomurai* tentacle autolysis solution to a depth of approximately 1 cm. To simulate natural envenomation, the skin was repeatedly bathed with the solution every 30 min using a plastic pipette. For the negative control (NC) group (*n* = 4), the same procedure was performed using seawater. After 8 h, the mice were anesthetized in a sealed chamber containing isoflurane (Merck, Darmstadt, Germany) and subsequently euthanized by cervical dislocation. Dorsal skin tissue samples were excised and fixed in paraformaldehyde. The fixed tissues were then sequentially dehydrated, embedded, sectioned, and baked for 2 h at 60 °C. The resulting sections were deparaffinized in xylene and rehydrated through a graded series of ethanol. To block non-specific background staining, endogenous peroxidase activity was quenched by incubating the sections with Endogenous Peroxidase Blocking Buffer (Beyotime, Shanghai, China) for 10 min, followed by three washes in PBS. The TE polyclonal antibody was diluted 1:1000 in Universal Blocking and Antibody Dilution Buffer (Beyotime). The sections were incubated with the primary antibody solution for 2 h at 37 °C. After washing, they were incubated with a goat anti-rabbit IgG-HRP secondary antibody (1:10,000) for 30 min at 37 °C. The sections were then visualized with a DAB kit (Beyotime) for 5 min, thoroughly rinsed with tap water, and counterstained with hematoxylin for 2 min. Finally, the sections were dehydrated and mounted with Neutral Balsam (Beyotime, Shanghai, China). Images were captured using a fluorescence microscope (Zeiss, Oberkochen, Germany) and analyzed with ImageJ software (v1.51, https://imagej.nih.gov/ij/, accessed on 8 February 2025).

### 2.7. Establishment of i-ELISA Assay

An indirect ELISA was developed to detect the TE antigen using the ELISA Basic Kit (Lianke Bio, Hangzhou, China). Briefly, 96-well polystyrene microplates (Corning, Shanghai, China) were coated with 100 μL/well of TE, which was serially diluted in coating buffer (Lianke Bio) to create a standard curve (0, 1, 2, 4, 8, 12, 16, and 20 ng/mL), and incubated overnight at 4 °C. The plates were then incubated for 3 h at room temperature. After washing three times with 300 μL/well of wash buffer (Lianke Bio), the plates were blocked by adding 250 μL/well of assay buffer (Lianke Bio) and incubating for 3 h at room temperature. Following another wash step, 100 μL of TE polyclonal antibody, diluted 1:10 in PBST (Yamei), was added to each well, and the plates were incubated for 23 h at 37 °C. After three washes, 100 μL of HRP-conjugated goat anti-rabbit IgG antibody (Lianke Bio), diluted 1:10,000 in dilution buffer (Lianke Bio), was added and incubated for 1 h at 37 °C. After a final series of three washes, the reaction was developed by adding 100 μL of TMB (3,3′,5,5′-Tetramethylbenzidine) substrate (Lianke Bio) and incubating for 15 min at room temperature in the dark. The reaction was terminated with stop solution (Lianke Bio). The absorbance was measured at 450 nm with a reference wavelength of 570 nm (OD450–570) using a microplate reader. The cut-off value for positivity was calculated as the mean ($\bar{X}$) plus three times the standard deviation (s) of the negative samples. A sample was considered positive if its OD450–570 value was ≥$\bar{X}$ + 3 s [21].

### 2.8. Precision Assessment of i-ELISA

To evaluate the precision of the established i-ELISA, eight different batches of TE were first quantified using a BCA Protein Assay Kit. The samples were then diluted to a final concentration of 10 ng/mL with coating buffer (Lianke Bio). The assay was performed according to the i-ELISA protocol described above. For intra-assay precision, each of the eight batches was assayed in quadruplicate on a single microplate. For inter-assay precision, the assay was conducted on four different microplates. The mean ($\bar{X}$) and standard deviation (s) of the OD450–570 values were calculated for both the intra- and inter-assay measurements. The coefficient of variation (CV) was determined using the formula: CV (%) = (s/$\bar{X}$) × 100. Both intra-assay and inter-assay CVs were calculated accordingly.

### 2.9. Detection of Residual Venom on Skin

To more closely simulate a natural sting event, 32 male ICR mice with depilated dorsal skin were divided into a negative control (NC) group and a Sting group. The skin was topically treated with seawater (NC group) or TE (Sting group) for 15 min. Subsequently, the residual liquid on the skin surface was collected by a cell scraper into 100 μL of coating buffer (Lianke Bio). The collected samples were then analyzed by the i-ELISA. Similarly, seawater and TE were applied to human skin for 15 min, and the residues were collected using the same scraping method for analysis. This human experiment, which posed no significant risk to the subject, was performed on one of the authors who had provided written informed consent.

### 2.10. Optimization of i-ELISA Conditions for Serum Residual Venom Detection

Serum dilution: Serum samples were collected from mice in the NC group (administered PBS via tail vein injection) and the *N. nomurai* group (treated with *N. nomurai* tentacle lysate for 8 h) and subsequently diluted with coating buffer (Lianke Bio) at ratios of 1:10, 1:100, 1:1000, and 1:10,000. 100 μL was added per well, and OD_450_–OD_570_ values for *N. nomurai* (P) and control group (N) serum were measured. The dilution with the highest P/N ratio was chosen as optimal.

Serum coating time: Using the optimal dilution, NC and *N. nomurai* group serum samples were coated at 4 °C for 1 h, 3 h, or 12 h. P and N values were measured, and the condition with the highest P/N ratio was selected.

Incubation time of goat anti-rabbit IgG-HRP: Under the previously optimized conditions, various incubation times (30 min, 60 min, and 120 min at 37 °C) were tested. P and N values were compared, and the time with the highest P/N ratio was defined as optimal.

Color development time: Under the optimized conditions above, tetramethylbenzidine (TMB) color development times of 5 min, 15 min, and 30 min were tested, followed by stop solution addition. OD_450_–OD_570_ was measured, and the optimal color development time was selected.

### 2.11. Sensitivity and Specificity of i-ELISA for Detecting Residual Venom in Serum

To evaluate the sensitivity and specificity of the established i-ELISA, mice were divided into four groups: a negative control (NC) group (administered PBS via tail vein injection, *n* = 72), a TE-IV group (intravenous injection of TE, *n* = 48), an *R. esculentum*-IV group (intravenous injection of *R. esculentum* venom, *n* = 21), and an *N. nomurai* group (topical envenomation with *N. nomurai* tentacle autolysis solution, *n* = 100). At 8 h post-treatment, serum samples were collected from all groups. The presence of residual venom (antigen) in the sera was then determined using the i-ELISA protocol with the anti-TE polyclonal antibody. Samples with OD_450_–OD_570_ ≥ $\bar{X}$ + 3s were considered positive. Sensitivity for the *N. nomurai* group was calculated as (number of positive serum samples in the *N. nomurai* group/total serum samples in the *N. nomurai* group) × 100%; TE-IV group serum sensitivity = (number of positive serum samples in the TE-IV group/total TE-IV group samples) × 100%; specificity = (number of negative samples in *R. esculentum*-IV and NC serum samples/total number of serum samples from *R. esculentum*-IV and NC groups) × 100%.

### 2.12. Statistical Analyses

All data are presented as mean ± SEM. Data were analyzed by one-way ANOVA, and the differences in *p* < 0.05 between groups were considered significant.

## 3. Results

### 3.1. Preparation of N. nomurai Venom Polyclonal Antibody

**Figure 1:**
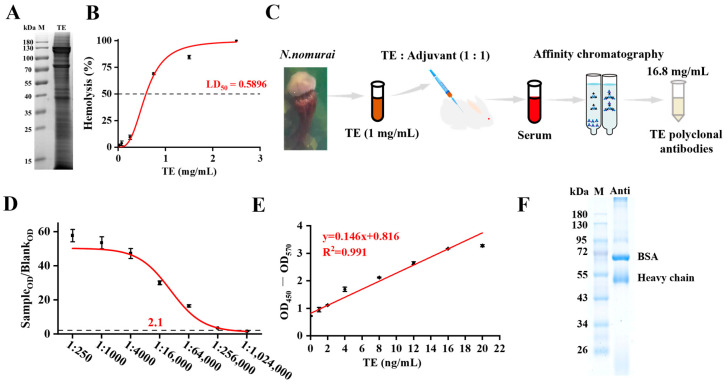
Preparation and titration of *N. nomurai* venom polyclonal antibody. (**A**) SDS-PAGE analysis of TE; (**B**) in vitro hemolytic activity of jellyfish TE, *n* = 6; (**C**) workflow diagram of TE polyclonal antibody preparation; (**D**) ELISA results of polyclonal antibody titration, *n* = 4; (**E**) linear relationship between jellyfish TE (as coating antigen) and polyclonal antibody (as detecting antibody), *n* = 4; (**F**) purity analysis of polyclonal antibodies, *n* = 4.

*N. nomurai* is one of the most commonly encountered and widely harmful toxic jellyfish along the coasts of China and worldwide. In this study, the protein distribution of the prepared *N. nomurai* tentacle venom (TE) was determined by gel electrophoresis. The analysis revealed a complex protein profile characterized by several prominent bands, including major bands with molecular masses of approximately 125, 75, and 38 kDa (Figure 1A). Furthermore, the TE exhibited potent hemolytic activity, with an LD50 value of 0.5896 mg/mL (Figure 1B). New Zealand white rabbits were immunized via multiple subcutaneous injections of small amounts of TE at multiple sites, and TE polyclonal antibody was subsequently purified from serum by chromatography (Figure 1C). ELISA titer determination showed that the obtained polyclonal antibodies had a titer of up to 1:256,000 (Figure 1D). i-ELISA analysis of antigen–antibody binding demonstrated a good linear relationship between coated antigen (TE) and the polyclonal antibody in the range of 0–20 ng/mL, with R^2^ = 0.992 (Figure 1E), suggesting a high affinity between TE antigen and antibody. In addition, The purified antibody exhibited a prominent band corresponding to the IgG heavy chain (50 kDa), with an overall purity estimated to be over 85%, thereby confirming its successful purification for immunoassay applications (Figure 1F). Collectively, these results indicate that the TE polyclonal antibody prepared in this study possesses strong affinity and provides a material foundation for the subsequent development of i-ELISA-based specific *N. nomurai* venom detection kits.

### 3.2. Establishment and Evaluation of the i-ELISA Detection Assay

**Figure 2:**
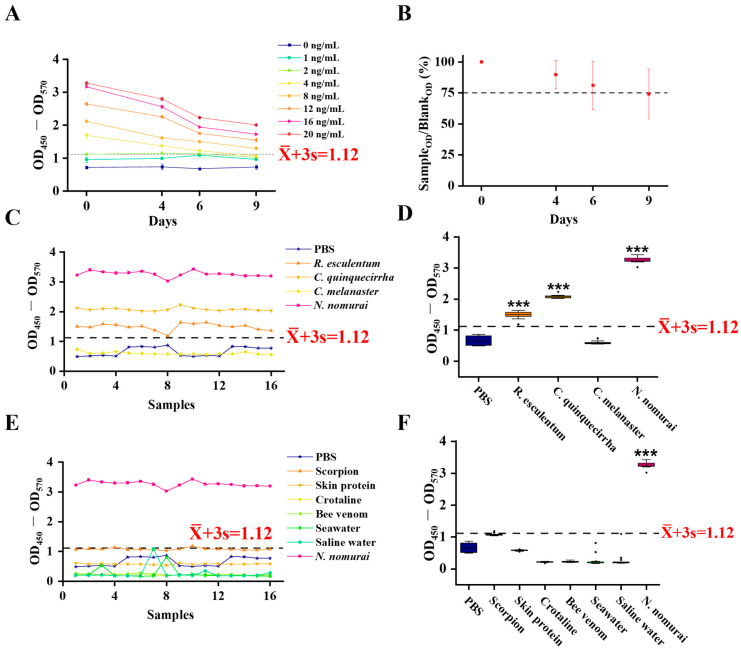
Establishment and evaluation of the i-ELISA assay. (**A**) Stability assessment at 37 °C, with measurements at days 4, 6, and 9, *n* = 4; (**B**) ratio of OD values measured at day 9 (incubated at 37 °C) to the baseline value, *n* = 4; (**C**,**D**) i-ELISA detection of TE (1 ng/mL, 10 ng/mL), *R. esculentum* venom (10 ng/mL), *C. quinquecirrha* venom (10 ng/mL), and *C. melanaster* venom (10 ng/mL), *n* = 4; (**E**,**F**) i-ELISA detection of TE (1 ng/mL, 10 ng/mL), scorpion toxin (10 ng/mL), melittin (10 ng/mL), phorbol ester, ICR mouse skin protein (10 ng/mL), seawater, and saline, *n* = 4. *** *p* indicates a significant difference (*p* < 0.001) compared with the PBS group.

Leveraging the high affinity of the polyclonal antibody for TE, an i-ELISA detection kit for *N. nomurai* venom was developed, comprising the anti-TE polyclonal antibody, a goat anti-rabbit IgG-HRP conjugate, TMB substrate, and a stop solution. The kit’s sensitivity, specificity, and stability were systematically evaluated. To assess stability, various concentrations of TE were measured using the established i-ELISA. Following the initial measurement (Day 0), the plate was stored in a sealed, dark environment at 37 °C and re-assayed on days 4, 6, and 9. The results showed that the initial limit of detection (LOD) for TE was 4 ng/mL. The LOD increased to 8, 8, and 12 ng/mL on days 4, 6, and 9, respectively, indicating excellent long-term stability (Figure 2A,B). Concurrently, the signal decay for 10 ng/mL TE was less than 15% after 6 days of storage at 37 °C, highlighting its robust stability. For specificity assessment, the kit was used to test venoms from *R. esculentum*, *C. quinquecirrha*, and *C. melanaster*. The assay demonstrated high specificity for *N. nomurai* venom, with low cross-reactivity towards venoms from other jellyfish genera (*Rhopilema*, *Chrysaora*, and *Cyanea*) (Figure 2C,D). Similarly, no positive signals were detected for scorpion toxin, melitracen, croton oil, ICR mouse skin protein, seawater, or saline (Figure 2E,F). This indicates that the kit lacks cross-reactivity with scorpion toxin and other unrelated substances, confirming its high specificity and robustness. The precision of the kit was evaluated using 10 ng/mL TE. Microplates were oscillated 10 times during each wash step to minimize CV. Results showed that intra-plate CV ranged from 0.77% to 2.78% (Table 1). The inter-plate CV was between 2.25% and 5.17% (Table 2), which meets the clinical standard (CV < 10%) and demonstrates robust batch-to-batch reproducibility. In summary, this ELISA kit combines high precision (4 ng/mL), strong specificity, and good stability, providing a reliable basis for clinical diagnosis and rapid field detection of *N. nomurai* venom.

### 3.3. Detection of Residual Venom on Skin by i-ELISA

**Figure 3:**
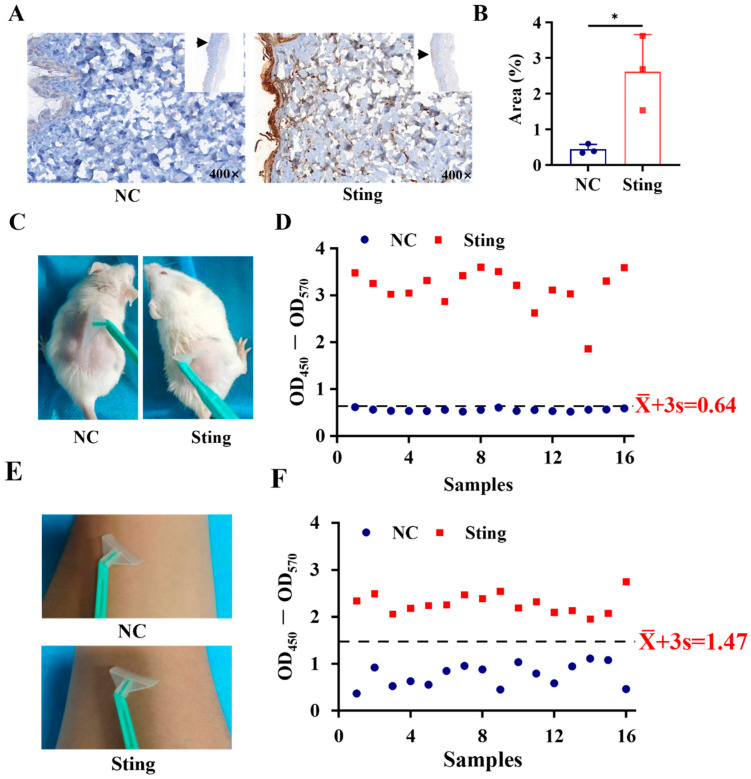
Detection of residual venom on skin by i-ELISA. (**A**) Immunohistochemistry of skin samples from NC and Sting groups, magnification 400×; (**B**) statistical analysis of immunohistochemistry in NC and Sting groups, *n* = 3; (**C**) dry seawater and TE collected from the dorsal skin surface of male ICR mice; (**D**) i-ELISA results for dry seawater and TE from ICR mouse skin surfaces; (**E**) dry seawater and TE collected from human skin; (**F**) i-ELISA results for dry seawater and TE collected from human skin. The data are presented as mean ± SEM, * *p* < 0.05.

Toxic organisms can release toxic or harmful substances through the skin, and toxin proteins may remain on the skin surface. Immunohistochemical results revealed marked and specific positive staining signals in ICR mouse skin after venom exposure, indicating the presence of residual TE on the skin surface (Figure 3A,B). i-ELISA detection of skin scrapings showed that the OD450–OD570 value in the TE exposure group was significantly higher than the positive threshold (0.64) (Figure 3C,D), confirming high sensitivity for detecting residual venom on skin. Similarly, positive detection of TE was achieved in residual fluids collected from the surface of human arm skin after venom application (Figure 3E,F), indicating the potential applicability in clinical samples.

In summary, consistent results from immunohistochemistry and ELISA confirm that the kit reliably detects residual venom on the skin and provides a reliable tool for the diagnosis of jellyfish stings.

### 3.4. Establishment and Optimization of Mouse Serum ELISA Diagnostic Method

**Figure 4:**
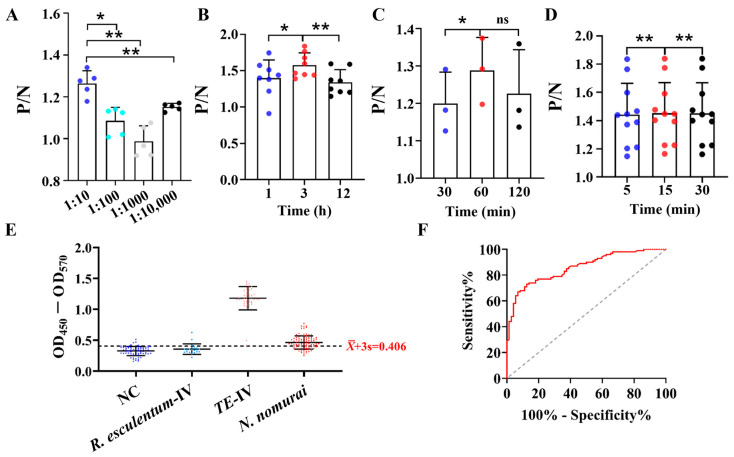
Optimization of ELISA parameters for serum from envenomed mice. (**A**) Optimal serum dilution, *n* = 5; (**B**) optimal serum coating time, *n* = 8; (**C**) optimal secondary antibody incubation time, *n* = 3; (**D**) optimal color development time, *n* = 11; (**E**) i-ELISA analysis of serum from the NC group (*n* = 72), *R. esculentum*–IV group (*n* = 21), TE–IV group (*n* = 48), and *N. nomurai* group (*n* = 100); (**F**) ROC analysis of serum i-ELISA for the NC and *N. nomurai* groups. The data are presented as mean ± SEM. * *p* < 0.05, ** *p* < 0.01.

Building upon the established i-ELISA detection method for *N. nomurai* venom, we further evaluated serum samples from mice after venom exposure. According to P/N ratios, the optimal serum dilution for diagnosis was determined to be 1:10 (Figure 4A), the optimal coating time was 3 h (Figure 4B), the optimal secondary antibody (goat anti-rabbit IgG-HRP) incubation was 1 h (Figure 4C), and the best TMB color development time was 30 min (Figure 4D). Using OD_450_–OD_570_ = $\bar{X}$ + 3 s as the threshold, a cutoff value of OD_450_–OD_570_ = 0.406 was set to distinguish positive and negative serum samples. The detection sensitivity for TE–IV group serum samples was 100%; for *N. nomurai* group serum, the sensitivity was 68.00% (95% CI: 57.99–76.95%) and the specificity was 90.32% (95% CI: 81.03–96.08%) (Figure 4E and Table 3). ROC analysis showed an AUC of 0.8596 (95% CI: 0.8015–0.9177; *p* < 0.001), indicating significant diagnostic discrimination. The optimal cut-off calculated by maximum Youden index was OD_450_–OD_570_ = 0.399, closely matching the preset threshold (0.406) (Figure 4F), further confirming the reliability of the cutoff.

In conclusion, the developed i-ELISA assay can also detect jellyfish venom in serum, providing a methodological basis for the development of diagnostic kits for venom exposure from jellyfish and other toxic marine organisms.

## 4. Discussion

Fatal envenomation by toxic jellyfish represents a growing global marine public health concern, with the rapid and accurate etiological diagnosis constituting a major bottleneck for effective clinical intervention and epidemiological surveillance. Currently, the diagnosis of jellyfish stings relies primarily on indirect evidence such as patient self-reporting, clinical symptomatology, and geographical information, lacking objective standards that directly detect the culpable agent, jellyfish venom, an issue similarly observed with snake envenomations [22]. In this study, we sought to establish a highly sensitive and specific immunoassay for the detection of *N. nomurai* venom, providing a crucial diagnostic tool for *N. nomurai* envenomation. The generation of high-affinity polyclonal antibodies was a key technical prerequisite [23]. The resulting antisera exhibited high titers (1:256,000), strong affinity, and high purity. Immunogenicity assessment via SDS-PAGE revealed multiple prominent venom protein bands, particularly at 125, 75, and 38 kDa, suggesting the presence of conserved or major antigenic components within the venom that could represent important targets for future antibody specificity improvement. The high-titer polyclonal antibodies demonstrated remarkable specificity for jellyfish venom, indicating the feasibility of antivenom development via host immunization as well as broader applications in rapid diagnostic kit design. Moreover, this study optimized and established an indirect ELISA (i-ELISA) for the detection of jellyfish venom. This assay allows for the detection of *N. nomurai* venom at concentrations as low as 4 ng/mL and maintains sensitivity after prolonged storage (at least 9 days at 37 °C), with projected stability for up to one year at 4 °C [24]. These findings highlight the practicality and portability of this method for clinical or environmental surveillance. The assay exhibited excellent reproducibility, with intra- and inter-assay coefficients of variation (CV) well within the acceptable range for clinical applications (CV < 10%) [25], ensuring reliability and batch-to-batch consistency. Importantly, the method distinctly differentiates *N. nomurai* venom from those of related species (e.g., *Rhopilema esculentum*, *Chrysaora quinquecirrha*, and *Cyanea melanaster*) as well as from various unrelated biological and environmental proteins. Indeed, Future research should certainly broaden the specificity testing to include a more comprehensive set of sympatric cnidarians, such as *Cyanea capillata* and *Aurelia aurita*, to fully validate its clinical utility in areas with greater species diversity. This specificity is critical for the accurate identification and diagnosis of *N. nomurai* stings, given the sympatric distribution and morphological similarities of jellyfish species in Chinese and global coastal waters.

Importantly, toxic antigens may persist on the skin following contact with venom [22]. Our assay demonstrated clear detection of venom residues on mammalian (murine and human) skin, with results highly consistent with those obtained via immunohistochemistry, further underscoring its practical utility in both clinical and experimental settings. In addition, effective detection of venom in serum represents a significant diagnostic approach [26]. Our assay discriminated between sera from animals systemically exposed to *N. nomurai* venom and control sera from unexposed animals, with an area under the curve (AUC) of 0.8596, indicating reliable diagnostic performance. The method achieved 100% sensitivity in the intravenous injection model and exhibited high specificity in the cutaneous envenomation model, further supporting its translational potential for clinical diagnostics. Collectively, these findings demonstrate the feasibility and analytical advantages of this immunoassay as an innovative platform for jellyfish venom exposure detection, addressing a critical need for objective diagnostic standards in jellyfish stings.

Certainly, several aspects remain open for future investigation. Firstly, while the polyclonal antibodies demonstrated excellent performance and reproducibility in the current study, the development of monoclonal antibodies targeting specific, stable, and abundant protein components within the venom—such as the 125 kDa or 75 kDa proteins identified herein—will undoubtedly enhance assay specificity and eliminate inter-batch variability. This will pave the way for the development of more robust and standardized diagnostic tools. Second, the reduced sensitivity of serum samples from the dermal envenomation model (68%) compared to the intravenous injection model (100%) likely reflects two key factors. Firstly, a high local retention of jellyfish venom within the cutaneous tissue upon administration may result in a diminished quantity of specific analytes entering the systemic circulation. Concurrently, venom components that enter the bloodstream undergo rapid dilution, systemic distribution, and subsequent metabolic clearance by organs such as the liver and kidneys. The 8-h exposure period in our experimental model provides an ample timeframe for the substantial clearance of venom from the circulation, thereby contributing to the lower detection sensitivity in the dermal model. Prospectively, the detection rate could be enhanced by adopting signal amplification technologies, such as the biotin-avidin system [27,28], or by developing assays that target more stable venom metabolites. This constitutes a critical direction for our subsequent investigations. Moreover, it is essential to recognize that this study was limited to simulated venom models. To translate this detection method from the laboratory to clinical practice, rigorous validation using a broad range of human clinical samples from actual sting cases is required. This poses ethical and logistical challenges but is an imperative next step. Additionally, the platform could be adapted into formats more suitable for point-of-care testing, such as lateral flow assays (LFA) [29], enabling real-time diagnosis of *N. nomurai* stings. In summary, this study systematically established and validated a highly sensitive and specific i-ELISA for the detection of *N. nomurai* venom. The assay demonstrated outstanding analytical performance in vitro, and its potential utility was further confirmed in simulated clinical scenarios using skin and serum samples.

This work provides a promising and reliable tool for the clinical diagnosis, forensic identification, and epidemiological study of *N. nomurai* envenomation, thereby laying a solid methodological foundation for the development of related diagnostic products and the optimization of future treatment strategies.

## 5. Conclusions

In this study, we successfully developed and rigorously validated a highly sensitive and specific indirect enzyme-linked immunosorbent assay (i-ELISA) for the detection of *Nemopilema nomurai* venom. The assay, predicated on a high-titer polyclonal antibody, demonstrates exceptional analytical performance, including high sensitivity (LOD: 4 ng/mL), robust specificity against venoms from other sympatric jellyfish species, excellent thermal stability, and high reproducibility (intra- and inter-assay CVs < 10%). Its practical utility was confirmed through the successful detection of residual venom in both cutaneous (murine and human) and systemic (murine serum) simulated envenomation models, exhibiting reliable diagnostic accuracy (AUC = 0.8596). In conclusion, this i-ELISA represents a significant advancement, providing a reliable and validated tool to address the critical gap in the etiological diagnosis of *N. nomurai* stings. It establishes a solid methodological foundation for the future development of point-of-care diagnostic kits and for optimizing clinical management and epidemiological surveillance of marine envenomations.

## Figures and Tables

**Table 1:** Intra-plate coefficient of variation, *n* = 4.

Sample	1	2	3	4	5	6	7	8
$\bar{X}$	3.8071	3.8099	3.7714	3.7621	3.7320	3.6748	3.5135	3.4996
s	0.0821	0.1058	0.0532	0.0473	0.0287	0.0827	0.0561	0.0610
CV (%)	2.1564	2.7770	1.4106	1.2573	0.7690	2.2505	1.5967	1.7431

Note: s: standard deviation; $\bar{X}$: mean; coefficient of variation (CV) = s/$\bar{X}$.

**Table 2:** Inter-plate coefficient of variation, *n* = 4.

Sample	1	2	3	4	5	6	7	8
$\bar{X}$	3.7360	3.6825	3.6721	3.6666	3.7018	3.6781	3.7200	3.7132
s	0.0840	0.1199	0.1193	0.1215	0.1915	0.1547	0.1797	0.1630
CV (%)	2.2484	3.2560	3.2488	3.3137	5.1732	4.2060	4.8306	4.3897

Note: s: standard deviation; $\bar{X}$: mean; coefficient of variation (CV) = s/$\bar{X}$.

**Table 3:** Sensitivity and specificity of ELISA for the detection of serum.

	NC	*R. esculentum*-IV	TE-IV	*N. nomurai*
Positive	6	3	48	68
Negative	66	18		32
Samples	72	21	48	100
Sensitivity			100%	68%
Specificity	90.32%			

## Data Availability

The data presented in the current study are available on request from the corresponding author.

## Abbreviations

The following abbreviations are used in this manuscript:

i-ELISA	Indirect enzyme-linked immunosorbent assay
TE	Tentacle extract
P	OD_450_–OD_570_ values for *N. nomurai*
N	Control group
TMB	Tetramethylbenzidine
ROC	Receiver Operating Characteristic
AUC	Area under Curve
CV	Coefficients of variation
